# Prevalence of Central Obesity among Adults with Normal BMI and Its Association with Metabolic Diseases in Northeast China

**DOI:** 10.1371/journal.pone.0160402

**Published:** 2016-07-28

**Authors:** Peng Zhang, Rui Wang, Chunshi Gao, Lingling Jiang, Xin Lv, Yuanyuan Song, Bo Li

**Affiliations:** Department of Epidemiology and Biostatistics, Jilin University School of Public Health, Changchun, Jilin, China; College of Tropical Agriculture and Human Resources, University of Hawaii, UNITED STATES

## Abstract

**Objectives:**

The present study aimed to investigate the prevalence of central obesity among adults with normal BMI and its association with metabolic diseases in Jilin Province, China.

**Methods:**

A population-based cross-sectional study was conducted in 2012 in Jilin Province of China. Information was collected by face to face interview. Descriptive data analysis and 95% confidence intervals (CI) of prevalence/frequency were conducted. Log-binomial regression analyses were used to find the independent factors associated with central obesity and to explore the adjusted association between central obesity and metabolic diseases among adults with normal BMI.

**Results:**

Among the adult residents with normal BMI in Jilin Province, 55.6% of participants with central obesity self-assessed as normal weight and 27.0% thought their body weight were above normal. 12.7% of central obesity people took methods to lose weight, while 85.3% didn’t. Female, older people and non-manual worker had higher risk to be central obesity among adults with normal BMI. Hypertension, diabetes and hyperlipidemia were significantly associated with central obesity among adults with normal BMI, the PRs were 1.337 (1.224–1.461), 1.323 (1.193–1.456) and 1.261 (1.152–1.381) separately when adjusted for gender, age and BMI.

**Conclusions:**

Hypertension, diabetes and hyperlipidemia were significantly associated with central obesity among adults with normal BMI in Jilin Province, China. The low rates of awareness and control of central obesity among adults with normal BMI should be improved by government and health department.

## Introduction

Obesity is a medical condition that body fat accumulates to a certain degree, which may have adverse effects on health, thereby reducing the life expectancy and health [1]. It is commonly caused by a combination of excessive intake of food energy, lack of physical activity and genetic susceptibility. Obesity is a leading preventable cause of death at present, with increasing rates in adults and children. In 2014, more than 1.9 billion adults aged 18 years and older were overweight. Of these over 600 million adults were obese. The worldwide prevalence of obesity more than doubled between 1980 and 2014 [2].

The traditional definition of “obesity” is based on body mass index (BMI). However, it can be further evaluated in terms of fat distribution via waist circumference (WC) and total cardiovascular risk factors [3, 4]. With the increase of overall WC, the risk of death also increased [5]. In a cohort study of the National Health and Nutrition Examination Survey (NHANES III), WC is proven to be more suitable to explain obesity-related health risks when metabolic syndrome was taken as an outcome measure [6]. In other words, excessive WC appears to be more of a risk factor for metabolic syndrome than BMI [7]. In recent years, many studies have been performed on the association between central obesity and metabolic diseases, but the data on the relationship among adults with normal BMI is relatively rare [8–11].

China is a large developing country in the world. The rapid development of economic has promoted the change of lifestyle such as dietary habit and physical activity [12]. Although the prevalence of obesity in China is lower than that of developed countries, overweight and obesity have become a major public health problem in China [13–15]. Jilin Province is located in the northeast of China, with a population of about 27 million [16]. Data in our study were obtained from the Jilin Provincial Chronic Disease Survey in 2012,which is the first large representative population-based survey of chronic disease in this area. In our study, we concentrate on the prevalence of central obesity and the association between central obesity and socio-demographic factors among adults with normal BMI. The distribution of attitude and behavior in normal and central obesity participants among adults with normal BMI were also described. Adjusted associations between central obesity and metabolic diseases among adults with normal BMI were explored in order to clarify the risk of metabolic diseases.

## Methods

### Subjects

A population-based cross-sectional survey was conducted among residents who were 18–79 years old and were living in Jilin Province for over six months in 2012. The multistage stratified cluster sampling method was used to select the study sample. Nine regions (Changchun, Jilin, Siping, Liaoyuan, Tonghua, Baishan, Songyuan, Baicheng and Yanbian), 32 districts or counties, 95 towns or communities, and 45 units in Jilin Province were selected. Details of the stratification process were reported previously [17]. 23,050 subjects aged over 18 years were recruited. 21,435 subjects completed the survey, resulting in a response rate of 84.9%. Response rates of urban and rural areas were 81.8% and 88.6%, respectively. A total of 9447 normal BMI people were chosen for the study.

### Ethical Standards

Ethical approval was obtained by Jilin University School of Public Health, and written informed consent was obtained from all subjects.

### Data Collection

The data of this survey is composed of three parts: questionnaire investigation (socio-demographic characteristics and health related information), body measurements (such as height, weight, WC and blood pressure) and laboratory measurements (such as serum cholesterol and triglyceride). All investigators were trained and they followed the same questionnaire instructions.

### Measurements

The height and weight of the subjects were measured without shoes. A calibrated mercury sphygmomanometer was used to determine the blood pressure of subjects on the right arm, after at least 5 minutes of seated rest. Blood pressure was measured three times with intervals of at least one minute, and we use the average value for data analysis. The investigator places the tape 0.5–1.0 cm above the navel level around a circle to measure WC. At the same time, the subjects were required to breathe naturally and wear thin clothes. The blood sample was obtained in the morning from subjects that were fasting for at least eight hours, and then conserved in tubes which contained EDTA [18].

### Definitions

According to the criteria of weight for Chinese adults [19], 18.5 ≤ BMI < 24 were defined as normal BMI,WC ≥ 80cm for female and WC ≥ 85cm for male were defined as central obesity. According to the criteria of the “Chinese Guidelines on Prevention and Treatment of Dyslipidemia in Adults” [20], diagnosed as hyperlipidemia by a physician and/or abnormal blood lipids (TC ≥ 5.18mmol/L or TG ≥ 1.70mmol/L or HDL-C < 1.04mmol/L or LDL-C ≥ 3.37mmol/L) were defined as hyperlipidemia. Self-reported Diabetes Mellitus (DM) and/or a fasting serum glucose level ≥ 7.0mmol/L were regarded as DM [21]. Self-reported hypertension and/or abnormal blood pressure (systolic ≥ 140mmHg or diastolic ≥ 90mmHg) were regarded as hypertension [22].

### Data Analysis

In order to make the sample representative of the population in Jilin Province, all data analyses were weighted by post-stratification adjustment according to the distribution of region, urban/rural, age, and gender groups in Jilin Province of China, 2010. The minimal data set can be find in S1 Table. Descriptive data analysis and 95% confidence intervals (CI) of prevalence/frequency were conducted. Log-binomial regression analyses were used to adjust for potential confounding factors and to find the independent factors associated with central obesity. Eight covariates were included in the regression model to study the associations between socioeconomic characteristics and central obesity among adults with normal BMI. We also explored the adjusted association between central obesity and metabolic diseases among adults with normal BMI by using log-binomial regression analyses. Data were analyzed by the complex sampling function of SPSS 22.0 or SAS 9.4, and *p*≤ 0.05 was considered to be statistically significant.

## Results

After complex weighted computation, this study included 9447 normal BMI (18.5–24) people, representative of the general Jilin Province normal BMI people aged 18 years and over by socio-economic characteristics (Table 1). In the study, the mean age was 46.23±13.77 years, 53.0% were male, 92.1% were Han Chinese and 53.8% from urban area. The majority of the subjects were between 25~54 years of age, 23.6% were aged between 35~44; 45.8% accepted a senior middle school education or higher; 56.6% were manual worker; 78.1% were married and 34.6% had a family per capita monthly income between 1000~1999 RMB.

**Table 1:** Socio-demographic characteristics among adults with normal BMI aged 18 and over in Jilin Province, China.

Characteristic	n	%
**Gender**		
Male	4444	53.0
Female	5003	47.0
**Area**		
Urban	5083	53.8
Rural	4364	46.2
**Ethnic**		
Han	8697	92.1
Minorities	750	7.9
**Age(year)**		
18~24	1583	16.8
25~34	1951	20.7
35~44	2227	23.6
45~54	1736	18.4
55~64	1212	12.8
65~79	738	7.7
**Education**		
Primary school and below	2222	23.5
Junior middle school	2900	30.7
Senior middle school	2464	26.1
College and above	1861	19.7
**Marriage**		
Married	7375	78.1
Unmarried	1554	16.5
Divorced	181	1.9
Widowed	337	3.5
**Family per capita monthly income(RMB)**		
<500	1509	16.1
500~999	1639	17.3
1000~1999	3272	34.6
2000~2999	1978	20.9
≧3000	1049	11.1
**Occupation**		
Manual worker	5349	56.6
Mental worker	2299	24.3
Retired	726	7.7
Unemployed or Others	1073	11.4

Note: Complex weighted computation was used in the statistical analysis.

Table 2 describes the estimated prevalence of central obesity among adults with normal BMI aged 18 and over by socio-demographic characteristics in Jilin Province, China. The estimated prevalence of central obesity in urban area was 7.7% (95%CI: 7.1–8.3), and rural area was 6.2% (95%CI: 5.7–6.8). The estimated prevalence of central obesity declined by education status: 4.2 (3.8–4.6) from primary school and below, 3.9 (3.5–4.3) from junior middle school, 3.4 (3.0–3.8) from senior middle school and 2.4 (2.1–2.8) from college and above. The estimated prevalence of central obesity maximize at 45~54 years old (3.3%, 95%CI: 3.0–3.6) and then declined by age. There were higher central obesity rate among people whose family per capita monthly income between 1000~1999 RMB (4.7%, 95%CI: 4.3–5.2), married (11.6%, 95%CI: 11.0–12.3), Han Chinese (13.0%, 95%CI: 12.3–13.8) and among people who engaged in manual work (6.5%, 95%CI: 6.0–7.1).

**Table 2:** Estimated prevalence of central obesity among adults with normal BMI by socio-demographic characteristics in Jilin Province, China.

Characteristic	Male (n = 4444)% (95%CI)[n]	Female (n = 5003)% (95%CI)[n]	Total (n = 9447)% (95%CI)[n]
**Area**			
Urban	7.5 (6.7–8.4)[334]	7.9 (7.1–8.7)[393]	7.7 (7.1–8.3)[727]
Rural	5.2 (4.5–6.0)[233]	7.1 (6.3–7.9)[353]	6.2 (5.7–6.8)[586]
**Ethnic**			
Han	11.9 (10.9–13.0)[530]	14.0 (13.0–15.1)[699]	13.0 (12.3–13.8)[1229]
Minorities	0.8 (0.6–1.2)[37]	0.9(0.7–1.2)[47]	0.9 (0.7–1.1)[84]
**Age(year)**			
18~24	0.9 (0.5–1.5)[41]	0.9 (0.5–1.6)[45]	0.9 (0.6–1.3)[86]
25~34	1.7 (1.3–2.3)[76]	1.7 (1.3–2.3)[86]	1.7 (1.4–2.1)[162]
35~44	2.3 (1.8–2.8)[101]	2.6 (2.2–3.0)[129]	2.4 (2.1–2.8)[230]
45~54	3.2 (2.7–3.7)[142]	3.4 (3.0–3.9)[169]	3.3 (3.0–3.6)[311]
55~64	2.7 (2.3–3.2)[120]	3.5 (3.1–4.0)[177]	3.1 (2.9–3.5)[297]
65~79	1.9 (1.6–2.4)[87]	2.8 (2.4–3.3)[140]	2.4 (2.1–2.7)[227]
**Education**			
Primary school and below	2.0 (1.7–2.5)[91]	6.1 (5.5–6.9)[307]	4.2 (3.8–4.6)[398]
Junior middle school	4.0 (3.4–4.7)[176]	3.8 (3.3–4.4)[190]	3.9 (3.5–4.3)[366]
Senior middle school	4.1 (3.5–4.8)[181]	2.8 (2.4–3.3)[141]	3.4 (3.0–3.8)[322]
College and above	2.7 (2.2–3.3)[119]	2.2 (1.7–2.7)[108]	2.4 (2.1–2.8)[277]
**Marriage**			
Married	11.0 (10.0–12.0)[489]	12.2 (11.3–13.2)[610]	11.6 (11.0–12.3)[1099]
Unmarried	1.2 (0.8–1.8)[52]	0.8 (0.5–1.4)[41]	1.0 (0.7–1.4)[93]
Divorced	0.2 (0.1–0.4)[11]	0.3 (0.2–0.5)[13]	0.3 (0.2–1.4)[24]
Widowed	0.3 (0.2–0.6)[15]	1.6 (1.3–2.0)[82]	1.0 (0.9–1.2)[97]
**Family per capita monthly income(RMB)**			
<500	1.8 (1.5–2.3)[81]	3.8 (3.2–4.3)[188]	2.9 (2.5–3.2)[269]
500~999	2.1 (1.7–2.6)[94]	2.8(2.3–3.3)[137]	2.5 (2.1–2.8)[231]
1000~1999	4.3 (3.7–5.0)[192]	5.1 (4.5–5.8)[256]	4.7 (4.3–5.2)[448]
2000~2999	2.9 (2.3–3.6)[129]	2.2 (1.8–2.7)[112]	2.6 (2.2–3.0)[241]
≧3000	1.6 (1.2–2.1)[71]	1.1 (0.8–1.4)[53]	1.3 (1.1–1.6)[124]
**Occupation**			
Manual worker	6.4 (5.6–7.2)[284]	6.6 (5.9–7.4)[330]	6.5 (6.0–7.1)[614]
Mental worker	3.6 (3.0–4.3)[160]	2.8 (2.3–3.4)[138]	3.2 (2.8–3.6)[298]
Retired	1.9 (1.5–2.3)[84]	2.5 (2.1–2.9)[124]	2.2 (1.9–2.5)[208]
Unemployed or Others	0.9 (0.6–1.3)[39]	3.1 (2.6–3.6)[154]	2.0 (1.8–2.4)[193]

Note: Complex weighted computation was used in the statistical analysis.

Table 3 describes the distribution of attitude and behavior among normal and central obesity adults with normal BMI in Jilin Province, China. Among central obesity people, 55.6% self-assessed as normal weight and 27.0% thought their body weight were above normal. 12.7% of central obesity people took methods to lose weight, while 85.3% didn’t.

**Table 3:** The distribution of attitude and behavior in normal and central obesity participants among adults with normal BMI.

Attitude and behavior	Normal, n(%)	Central obesity, n(%)
**Self-assessment of body weight**		
Subnormal weight	2575(31.6)	228(17.4)
Normal weight	4123(50.7)	730(55.6)
Above normal weight	1436(17.7)	355(27.0)
**Weight control methods**		
Lose weight	797(9.8)	166(12.7)
Gain weight	289(3.6)	27(2.0)
No method	7048(86.6)	1120(85.3)

Note: Complex weighted computation was used in the statistical analysis.

Table 4 describes the association between central obesity and socio-demographic factors among adults with normal BMI aged 18 and over in Jilin Province, China. Female had higher risk to be central obesity (PR: 1.377, 95%CI: 1.251–1.518). Participants aged 35~79 were more likely to be central obesity compared with 18~24 years old (35~44 years old (PR: 1.545, 95%CI: 1.026–2.389), 45~54 years old (PR: 2.805, 95%CI: 1.876–4.311), 55~64 years old (PR: 4.163, 95%CI: 2.780–6.416), 65~79 years old (PR: 4.894, 95%CI: 3.226–7.621)). Similarly, non-manual workers were more likely to be central obesity compared with manual workers (mental worker (PR: 1.258, 95%CI: 1.070–1.476), retired (PR: 1.238, 95%CI: 1.213–1.571), unemployed or others (PR: 1.377, 95%CI: 1.251–1.518)). Area, ethnic, education level, marriage status and family per capita monthly income were not associated with central obesity.

**Table 4:** Association between central obesity and socio-demographic factors among adults with normal BMI in Jilin Province, China.

Characteristic	*p*	PR	95%CI
**Area**			
Rural		1	
Urban	0.581	0.975	0.876–1.085
**Gender**			
Male		1	
Female	**<0.001**	1.377	1.251–1.518
**Ethnic**			
Minorities		1	
Han	0.147	0.873	0.721–1.041
**Age(year)**			
18~24		1	
25~34	0.323	1.238	0.819–1.915
35~44	**0.043**	1.545	1.026–2.389
45~54	**<0.001**	2.805	1.876–4.311
55~64	**<0.001**	4.164	2.780–6.416
65~79	**<0.001**	4.894	3.226–7.621
**Education**			
Primary school and below		1	
Junior middle school	0.613	1.032	0.913–1.165
Senior middle school	0.710	0.973	0.842–1.123
College and above	0.997	1.000	0.818–1.218
**Marriage**			
Married		1	
Unmarried	0.123	0.769	0.542–1.059
Divorced	0.662	0.924	0.629–1.282
Widowed	0.654	1.037	0.881–1.209
**Family per capita monthlyincome (RMB)**			
<500		1	
500~999	0.820	1.016	0.883–1.168
1000~1999	0.970	0.997	0.873–1.140
2000~2999	0.135	1.137	0.960–1.343
≧3000	0.201	1.145	0.927–1.404
**Occupation**			
Manual worker		1	
Mental worker	**0.006**	1.258	1.070–1.476
Retired	**<0.001**	1.238	1.213–1.571
Unemployed or Others	**<0.001**	1.377	1.251–1.518

Note: PR = prevalence ratio; CI = confidence interval; Complex weighted computation was used in the statistical analysis.

As showed in Table 5, hypertension, diabetes and hyperlipidemia were significantly associated with central obesity, the PRs were 1.577 (1.434–1.735), 1.485 (1.321–1.659) and 1.530 (1.392–1.684) separately in Model 1. When adjusted for BMI in model 2, the effect values decreased. The PRs were 1.337 (1.224–1.461), 1.323 (1.193–1.456) and 1.261 (1.152–1.381), separately. We got a very similar result in Model 3, the PRs were 1.327 (1.215–1.451), 1.302 (1.181–1.430) and 1.251 (1.144–1.371), separately.

**Table 5:** Adjusted association between central obesity and metabolic diseases among adults with normal BMI in Jilin Province, China.

Diseases	Model 1^a^			Model 2^b^			Model 3^c^		
	*p*	PR	95%CI	*p*	PR	95%CI	*p*	PR	95%CI
**Hypertension**									
No		1.000			1.000			1.000	
Yes	<0.001	1.577	1.434–1.735	<0.001	1.337	1.224–1.461	<0.001	1.327	1.215–1.451
**Diabetes**									
No		1.000			1.000			1.000	
Yes	<0.001	1.485	1.321–1.659	<0.001	1.323	1.193–1.456	<0.001	1.302	1.181–1.430
**Hyperlipidemia**									
No		1.000			1.000			1.000	
Yes	<0.001	1.530	1.392–1.684	<0.001	1.261	1.152–1.381	<0.001	1.251	1.144–1.371

^a^ Model 1 adjusted for gender and age.

^b^ Model 2 adjusted for gender, age and BMI.

^c^ Model 3 adjusted for gender, age, BMI and occupation.

Note: PR = prevalence ratio; CI = confidence interval; Complex weighted computation was used in the statistical analysis.

## Discussion

Our study was a large population-based survey to investigate the prevalence of central obesity and the association between central obesity and metabolic diseases among adults with normal. Understanding the features of central obesity among adults with normal BMI can provide a new perspective for prevention of metabolic diseases: pay attention to your WC even you have a normal BMI. Most of the people with normal BMI believed that their body weight would not be a risk factor for diseases. In this study, there were just 27.0% of the participants with central obesity self-assessed their body weight as “above normal”, moreover, just 12.7% of them took methods to lose weight. It means that most of the participants with normal BMI thought they were far from the danger caused by obesity. Lack of understanding of obesity, especially central obesity, has made people not to take adequate measures to control obesity. Low awareness rate of central obesity in China was also reported by other research. *Sidney et at* reported that 23% of the general population were aware of the risk of central obesity, and just 6% of them having had WC measured by doctor or nurse in China [7]. Therefore, we suggest that the government and health department should take measures to improve people's awareness of central obesity in Jilin Province of China, especially the normal BMI population, as they are more likely to ignore the risk of central obesity.

The association between central obesity and socio-demographic was showed by log-binomial regression analyses. From 35 to 79 years old, the risk of central obesity increased gradually. As people get older, most of the body functions begin to decline gradually, as well as body metabolism level. Physical and mental consumption is reduced, while the corresponding reduction in dietary intake is not much. Besides, the distribution of fat in the body is also changing after middle age: body fat is more likely to accumulate in abdomen [23, 24]. Therefore, with the growth of age, the risk of central obesity increased gradually. Manual workers were less likely to be central obesity than other occupation status as they consumed more calories during work. A meta-analysis study showed that the combination of physical activity and dietary interventions can effectively reduce WC [25]. It’s necessary for central obesity people to reduce calorie intake and increase the amount of exercise. Physical activity may increase insulin sensitivity, glucose disposal, and oxidation of free fatty acids, which may reduce the complications of central obesity [26]. We explored the association between central obesity and metabolic diseases among adults with normal BMI in three models. Both of them indicated that hypertension, diabetes and hyperlipidemia had a positive association with central obesity, which is consistent with previous studies [27, 28]. Whether adjusting for BMI has a great impact on the results. One possible explanation for this is that BMI is very highly correlated with WC. The risk of metabolic diseases can be more accurately predicted by WC after adjustment for the influence of BMI. Other covariates were also examined and found not to be important to the result. Therefore, the covariates contained in model 3 were age, gender, BMI and occupation. Persons with normal BMI, but who had increased WC, have higher risk of metabolic diseases. Thus, blood glucose, blood lipid and blood pressure should be early detected among adults with normal BMI in order to reduce the prevalence of hypertension, diabetes and hyperlipidemia.

The strength of this study lies in a representative sampling survey based on a large population. Complex weighted computation was used in the statistical analysis, which increased the representativeness of our results. However, the study still has limitations. First, our sample excluded those who were ill or too weak to complete the interview. Second, the participants were recruited from Jilin Province of China, so the conclusion cannot represent the situation in other regions of China.

## Conclusions

Hypertension, diabetes and hyperlipidemia were significantly associated with central obesity among adults with normal BMI in Jilin Province, China. Our study highlighted the low rates of awareness and control of central obesity among adults with normal BMI. Efforts should be made to improve people's awareness of central obesity among adults with normal BMI by the government and health department.

## Supporting Information

S1 Table Minimal data set. (XLSX)
